# Single-Parent Families, Educational Gradient, and Child Deprivation: The Cases of Italy and Spain

**DOI:** 10.1007/s12187-022-09931-7

**Published:** 2022-03-07

**Authors:** Antonio L. Pérez-Corral, Almudena Moreno Mínguez

**Affiliations:** Department of Sociology and Social Work, University of Valladolid, Plaza de La Universidad, Campus María Zambrano, 40005 Segovia, Spain

**Keywords:** Child deprivation, Inequality, Single-parent families, Educational level, Southern Europe

## Abstract

This work examines whether the increase of single parenthood in Italy and Spain, specifically amongst women in an unfavourable socioeconomic position, has repercussions for child well-being, understood here as material deprivation. In particular, our main objective is to analyse the possible differential impact of single parenthood on children’s material deprivation in relation to mothers’ level of education. Using the 2014 EU-SILC Module on material deprivation, we identify five areas of child deprivation based on the EU-MODA approach: nutrition, clothing, education, leisure, and social life. In the case of Italy, our main results indicate that, compared to children from two-parent households, children of single mothers with a low level of education have a higher risk of nutrition and clothing deprivation. In Spain, living in a single-parent household is associated with a higher risk of deprivation in terms of social life for those children whose mothers do not have a high level of education. Therefore, the findings suggest that in both countries the growth of single parenthood amongst women with a lower educational level may have an impact on child well-being inequality. This article contributes empirical data to the growing literature on the rise of child poverty in Southern European countries.

## Introduction

Scientific literature has focused especially on the economic perspective of child well-being, attempting to measure it through different dimensions of child poverty (Bradshaw, 2015; Dijkstra, 2009; Gordon & Nandy, 2012; OECD, 2013). Traditionally, family income has been commonly used as an indicator of children’s economic well-being, although alternative indicators that better reflect children’s quality of life, such as material deprivation, are now being used more frequently (Ferrão et al., 2021; Guio et al., 2018; Main & Bradshaw, 2012). This growing interest in child well-being has taken place gradually and in parallel with demographic, family, and socioeconomic changes that have diversified family structures and increased family inequality and precarity (McLanahan, 2004; Nieuwenhuis & Maldonado, 2018).

Literature has viewed family structure as a fundamental factor in child well-being and family inequality (McLanahan, 2004; Rees et al., 2011). The increase in family diversity is characterized by the emergence of family structures such as single-parent families, where vulnerability increases due to the existence of a single economic provider. Likewise, these social and family transformations address the mother's education as a critical factor of inequality in transmitting the economic, cultural, and social disadvantages and advantages to her children (Garriga et al., 2015). Social stratification is essential in family research, although few studies have analyzed the association between single-parent families, inequality, and child deprivation in southern European countries (Moreno Mínguez & Dueñas, 2019). This model of analysis has been widely motivated in the Anglo-Saxon field of family studies, according to which there is a clear relationship between social structure, single parenthood, parenting, and child inequality (Berger & McLanahan, 2015; Carlson & McLanahan, 2004; Lareau, 2011).

The increase of the number of single parent families is explained within the interpretative framework of the Second Demographic Transition (SDT), according to which processes of individualisation and cultural change have led to the proliferation and diversification of new types of households in the second half of the twentieth century (Lesthaeghe, 1995, 2010; Martin & Kats, 2003; Van De Kaa, 1987). This interpretative paradigm has given rise to criticism by researchers of family sociology and demography (Esping-Andersen & Billari, 2015), as growing precarity and greater inequality call into question Lesthaeghe’s (2010) convergent tendencies. In this sense, the study of parents’ educational profile represents a suitable analytical tool for the analysis of the changes produced by the effects of SDT (diversification of family structures) on the growing inequality between families and subsequently on children’s material deprivation.

The empirical evidences suggest that the processes associated with this transition are leading to diverse educational and employment trajectories that have several consequences for child well-being (Chan & Halpin, 2008; Garriga et al., 2015; McLanahan, 2004). While parents with greater resources follow more successful routes that benefit their children, those who have limited means pursue alternative disadvantageous and precarious paths that are even more frequent in female-headed single-parent households, thus further expanding the gap between families (McLanahan, 2004). Subsequently, children of mothers from disadvantaged backgrounds experience deprivation that conditions the course of their life, further aggravating problems in single-parent homes. Therefore, one of the effects of SDT is the increase of inequalities in relation to child deprivation. However, the negative effects of single parenthood are not only defined by the educational level of the parents, but also by the effect of social policies which look to correct these imbalances (Nieuwenhuis & Maldonado, 2018). Recent studies have indicated that, after the Great Recession, inequality between family types increased in southern European countries (Mazzucchelli & Parise, 2019; Pérez Corral & Moreno Mínguez, 2021), where there are few specific policies to support single-parent families (Almeda et al., 2016; Nieuwenhuis & Van Lancker, 2020).

Based on this empirical evidence, the aim of this paper is to contribute to the existing literature on single parenthood and child well-being in southern Europe. To do this, we examine whether the effect of single parenthood on child deprivation varies according to the educational level of mothers in Spain and Italy. We focus on these countries in south Europe because, even though changes in the socioeconomic profile of single-parent families and in the social welfare state seem to have evolved in similar ways, results from previous studies also suggest that there are some differentiating nuances (Garriga & Cortina, 2017; Garriga et al., 2015; Moreno & Marí-Klose, 2013; Naldini & Jurado, 2013). Establishing what these differences might consist in may shed light on the design of family policies aimed at single-parent families and children.

## Single parenthood, family change and child well-being

The SDT has been associated with family and culture changes such as the increase in couple cohabitation, divorce, and births outside marriage which have favoured the growth of single-parent families, particularly those headed by women (Lesthaeghe, 2010). However, these changes have not evolved at the same rate in all European countries, and it is in the south of the continent where they are occurring at a slower pace, as the fertility and gender equality indicators show (Esping-Andersen & Billari, 2015; Hantrais, 2005). In this demographic and cultural context of reduced fertility and dominance of the male breadwinner family model (Pfau-Effinger, 2017), single-parent families are less frequent in Italy and Spain compared to Central and North European countries (Bernardi et al., 2018; Iacovou & Skew, 2010). This lower incidence of single parenthood is principally due to cultural and institutional factors of Mediterranean welfare states characterised by the dominance of family and traditional structures and the lack of family and employment policies orientated towards the needs of single-parent mothers (Almeda et al., 2016; León & Migliavacca, 2013; Naldini & Jurado, 2013). Comparative studies usually include Italy and Spain within the same welfare state regime, so few studies have focused on analysing the differences between the two countries and the effects of these differences on families, inequality, and child well-being.

**Fig. 1:**
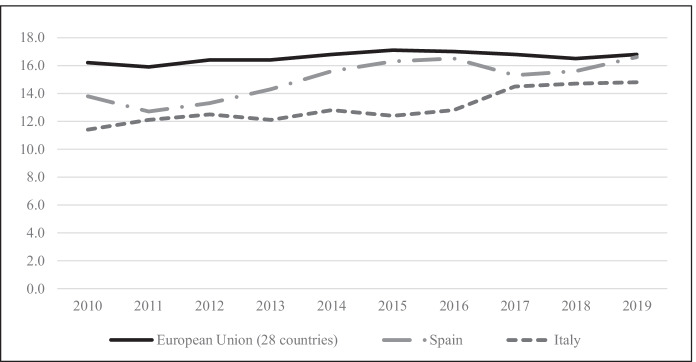
Percentage of children in single-parent households, 2010–2019. Source: Eurostat (Population and social conditions)

If the special characteristics of Mediterranean societies have slowed down family transformation in Italy and Spain, recent works indicate that the percentage of single-parent families is continuously growing in both countries (Nieuwenhuis, 2020; Nieuwenhuis & Maldonado, 2018). As Fig. 1 shows, during the last 10 years, there has been a general increase in the percentage of children living in single-parent households in both Italy and Spain. Despite commonalities between southern European countries, differences are also observed in terms of the degree of acceptance of new forms of family life, with Spain showing a greater degree of acceptance, while in Italy attitudes to these changes are more conservative (Moreno & Marí-Klose, 2013). In the same line of interpretation, several research works have highlighted the existing differences in attitudes and models of family and work reconciliation in Italy and Spain, thus putting into question the supposed uniformity between welfare states in the south of Europe. Specifically, comparative studies have shown that although family policies are more limited in both countries than in Northern Europe, Spain has developed a family policy that is more favourable to equality and work-family compatibility than Italy (Crespi & Moreno Mínguez, 2017; Naldini & Jurado, 2013).

The analysis of the incidence of single parenthood is crucial, as it is associated with precarity and economic disadvantage, leading to negative repercussions in child well-being. As previous evidence shows, children who live in single-parent homes are more likely to be affected by educational, social, and behavioural problems (Amato & Cheadle, 2005; Astone & McLanahan, 1991; Heintz-Martin & Langmeyer, 2020; Pong et al., 2003; Waldfogel et al., 2010). Garriga et al. (2015) indicate that in the mid-1990s, the differences in poverty ratios between single-parent and two-parent families were smaller in southern European countries than in most European countries, mainly due to the composition effect of single-parent families. Unlike the countries of northern and central Europe, there was a positive educational gradient on single parenthood in the southern countries (Garriga & Cortina, 2017; Kennedy & Thomson, 2010; Mclanahan, 2004). Therefore, the comparison of the socio-economic conditions of children in Europe cannot be adequately understood without considering the socio-demographic composition of family structures and the evolution of family typologies in each country. The parents’ education profile is an essential factor for child well-being, given that it influences parents’ employment opportunities and families’ economic situations (Garfinkel & Pilkauskas, 2016; Van Damme et al., 2009), as well as having a positive impact on the time that parents dedicate to their children (Gimenez-Nadal & Molina, 2013; Guryan et al., 2008). All of the above has a bearing on the subjective well-being of children and the development of skills through socialization (Ridge, 2007; Ridge & Millar, 2011). According to the Family Stress Model, the economic problems faced frequently by the most disadvantaged families impact the increase in parental stress, which affects the quality of upbringing and well-being of children (Conger & Elder, 1994; Conger et al., 2002; Zhang et al., 2020). In fact, considering that the educational level of the parents is usually used as an indicator of the socioeconomic status of the families (Mandemakers & Kalmijn, 2014), several studies have suggested that social class establishes differences in the practices of parents and parent–child interactions regarding the way they spend time (Kohn, 1963; Kohn & Schooler, 1983; Lareau, 2002; Weininger & Lareau, 2009). Lareau (2011) states that middle-class parents spend more time with their children doing activities that benefit child development than parents of lower socioeconomic origin. In sum, the results of previous research concluded that social class contributes to shaping different parental styles, through which economic, social, and cultural advantages and disadvantages are transmitted intergenerationally.

These findings are of interest to our study since they allow us to properly motivate and contextualize the interaction between the mother's education, family stratification, parental involvement, and child deprivation in single-parent families. This introduces a broader approach than previously discussed in the analyses of child poverty. In addition, it is necessary and appropriate to apply these analytical models to the Spanish and Italian cases, given the lack of comparative empirical evidence for these countries that may indicate for the need to design specific family policies aimed at single-parent families.

## Interpretative and Hypothetical Model

On the basis of previous considerations, the idea that the education of mothers at the head of single-parent households is a key variable for the study of child deprivation, understood as a lack of goods and services necessary for the well-being and correct development of children (Bárcena-Martín et al., 2017a; Chzhen & de Neubourg, 2014), is reinforced. Moreover, it is also relevant for establishing the existing differences between Italy and Spain. Studies have found that at the end of the twentieth century the education gap was not particularly relevant for single-parent compared to two-parent families in either country (Albertini & Dronkers, 2009; Garriga & Cortina, 2017; McLanahan, 2004). Nevertheless, these educational attainment differences have varied in recent decades. The findings of the study by Garriga et al., (2015) indicate that in 2005 as well as in 2011, the probability of living in single-parent households was greater for mothers with a lower educational level in Spain. In the case of Italy, while the study results show that in 2005 single mothers still had an educational advantage with respect to mothers living in two-parent households, this difference appears to have subsequently disappeared. In a later work, Garriga and Cortina (2017) examine in detail the evolution of educational attainment for single-parent mothers in Spain. One of their most significant results was finding that in 1991 there was an unequivocal positive relationship between single parenthood and maternal educational attainment which became less evident in 2001 and negative in 2011. In general, previous research shows a change in the educational level of single-parent mothers in Italy and, above all, Spain. In fact, in both countries there is a clearly negative educational gradient for the youngest single-parent mothers, thus pointing to the probability that changes in mothers’ educational differences according to family structure may become even more accentuated in the future (Garriga & Cortina, 2017; Garriga et al., 2015).

Studies available for the contexts of Europe and the USA have shown that negative consequences of single parenthood on child well-being are more pronounced in families where the mother has a low educational level (Albertini & Dronkers, 2009; Augustine, 2014; Grätz, 2015; Mandemakers & Kalmijn, 2014). However, there are other works that suggest results to the contrary (Bernardi & Boertien, 2016, 2017). In summary, previous empirical evidence shows that the differential effect of family type on child well-being according to the educational attainment of parents seems to be influenced by multiple factors such as, for example, the indicator of well-being under analysis or the country being studied (Bernardi & Boertien, 2017; Garriga & Berta, 2018; Grätz, 2015; Mandemakers & Kalmijn, 2014). In this sense, in countries where there is greater protection against poverty and precarity, single parenthood may not have a big impact on the economic situation of families with less educational attainment (Garriga & Berta, 2018; Leopold & Leopold, 2016). In consequence, in order to better understand the effect of educational gradient on child inequality, more empirical evidence and indicators that analyse different contexts must be taken into account.

Focusing on the Italian and Spanish cases, the main objective of this article is to study the differential effect of single parenthood on child deprivation according to the maternal educational profile. In order to do this, we shall analyse the impact on different types of deprivation as established by the Multiple Overlapping Deprivation Analysis for the European Union (EU-MODA). More specifically, EU-MODA distinguishes between types of deprivation by their relation with specific dimensions of child well-being (Chzhen & de Neubourg, 2014; Chzhen et al., 2016). Therefore, in this paper we will apply these indicators of child well-being to the analysis while comparing Italy and Spain as examples of Mediterranean welfare states.

It is worth pointing out that in southern European countries the differences in economic resources between single and two-parent families are smaller than in other countries in Europe, which is mainly due to the family support network for single-parent mothers and a greater prevalence of two-parent households with only one breadwinner (Hampden‐Thompson, 2009). Hence, the effect of family structure on child deprivation is usually less evident in countries such as Italy and Spain (see, for example, Guio et al., 2020). Nevertheless, we must take into consideration the fact that the Great Recession has had a greater impact on southern European countries, provoking an intense deterioration in employment lasting beyond the recuperation period (Duell et al., 2016; OECD, 2018) and in whose aftermath the risk of material deprivation in single-parent families may have increased (Nieuwenhuis & Maldonado, 2018). In this regard, it is to be expected that single-parent families headed by mothers with a low level of education are more affected by a context of greater economic and employment precarity than two-parent families with the same educational level, given that not having high qualifications may accentuate the disadvantages that single-parent mothers must confront in the job market as a consequence of their greater difficulties in conciliating work and family life (Härkönen et al., 2016). Moreover, for single-parent mothers with a low educational level, economic support from their family may not be enough to compensate a large part of the differences in resources in comparison with two-parent families, given that such help tends to be smaller in families of low socioeconomic standing (Eggebeen & Hogan, 1990; Majamaa & Rantala, 2019). Therefore, the growth of single parenthood among women with low educational attainment in southern Europe could contribute to inequality between family types in terms of child deprivation.

As a final hypothesis, we must add to all of the above that the impact of single parenthood on child well-being in relation to the mother’s educational level may not be the same in Italy and Spain due to the different family and work reconciliation policies existent in both countries, as confirmed in previous studies (Crespi & Moreno Mínguez, 2017; Naldini & Jurado, 2013). Specifically, these policies have made greater progress in the Spanish case, although they have been directed mainly to the needs of dual-earner families, paying less attention to the problems of reconciliation of single-parent families (Almeda et al., 2016). However, it is also true that reconciliation policies, even if they are not focused on single-parent families, can benefit, for example, divorced mothers by linking them to the labour market before separation (Maldonado & Nieuwenhuis, 2020). These differences between the two countries may mean that in Spain, single-parent mothers with a lower educational level can provide greater protection to their children against material deprivation than in Italy.

## Data, Variables and Methodology

### Data Set and Sample

The data for this study was obtained from the European Union Statistics on Income and Living Conditions (EU-SILC) (Eurostat, 2021). We work with cross-sectional microdata included in the 2014 wave, this being the latest available wave that includes a module with information on child material deprivation. While there is a similar module in the 2009 wave, some of the deprivation variables for 2014 present changes (European Commission, 2014a), thus making it difficult to use both waves together. In addition, focusing on 2014 allows us to consider the material and economic situation of families after the Great Recession.

We selected the samples of households with children from EU-SILC for Italy and Spain. Specifically, we include two-parent families and single-parent families headed by mothers.^1^ Moreover, we have restricted these samples to households where there is at least one child aged between 1 and 15 years. This is because child deprivation questions only take into consideration children of this age range (European Commission, 2014a). It should also be pointed out that deprivation questions refer to all children of the household jointly, as in this survey it is assumed that if one child lacks a certain item, the rest of the children also lack access to it.^2^ The final sample was made up of 4160 Italian and 3066 Spanish households.

### Child Deprivation

The perspective used in this article for measuring material child deprivation is based on EU-MODA, which is an adaptation of the UNICEF’s MODA methodology for the European context using EU-SILC data (Chzhen & de Neubourg, 2014). EU-MODA is a method for studying child poverty whose scope spans from the evaluation of deprivation rates in individual items to the analysis of the overlap between various measures of poverty (see Chzhen et al., 2016; Chzhen & de Neubourg, 2014; De Neubourg et al., 2012). From this ample procedure, we only use the aggregate of items in dimensions as per their relation with concrete aspects of child well-being. Each of these dimensions stands for one of our dependent variables for child deprivation. In general, the majority of studies on European level usually aggregate all the child deprivation items provided by the EU-SILC into one sole variable, which facilitates the interpretation of deprivation as well as comparison between countries, years or collectives (Bárcena-Martín et al., 2017a, b; Guio et al., 2020). Despite this, we have chosen to distinguish between different types of child deprivation, as this allows us to take into account the multidimensional nature of deprivation and to identify areas that may be more affected by the increase of single parenthood amongst women with a lower educational level in Italy and Spain. In reference to this, Heflin et al. (2009) emphasize that making such a distinction is apt, given that the causes of every type of material difficulty, as well as their impact on well-being, are not necessarily identical.

EU-MODA includes seven dimensions of deprivation of which we have omitted two (Information and Housing) due to the fact that these do not reflect the specific deprivation of children (Stefánsson et al., 2018). The dependent variables which we use in this study are related to the deprivation in the dimensions of nutrition, clothing, education, leisure, and social life of the children. We have followed the same item aggregation as that of Chzhen et al. (2018), who also use the 2014 deprivation module of EU-SILC. Nutrition was measured by two items: (a) fruits and vegetables once a day; and (b) one meal with meat, chicken, or fish at least once a day. Clothing was measured by two items: (a) some new clothes, and (b) two pairs of properly fitting shoes. Education was measured by three items: (a) books at home suitable for children's age, (b) attending childcare services for at least one hour a week, and (c) participate in school trips and events that cost money. Leisure was measured by three items: (a) outdoor leisure equipment, (b) indoor game, and (c) regular leisure activity. Social life was measured by two items: (a) celebrations of special occasions, and (b) invite friends to play or eat. All of the dimensions are similar for households with children aged between 1 and 15, except the education dimension. In those households where all the children are younger than three years, the deprivation in education is based only on the availability of books adequate for their age. In households where the children are older than two years but still have not reached the mandatory school age,^3^ children’s attendance to nurseries is also taken into consideration.^4^ For older children, the items used are the availability of books and participation in school trips.

Each of the dimensions are measured as binary variables that have a value of 1 if the children of the household are experiencing deprivation and 0 if not. In accordance with EU-MODA’s union approach, children’s deprivation in only one item is sufficient to consider that they are deprived in the corresponding dimension (Chzhen & de Neubourg, 2014). Deprivation is based on children’s inability to access a determined item, independently of the reason why the item may be unavailable.

### Explanatory Variables

The first of our independent variables reflects the household family structure, making a distinction between two categories: two-parent families and single-parent families headed by mothers. The variable does not identify single-parent families headed by fathers since they are not included in our sample. There are too few observations to analyse their relationship to child deprivation. Although EU-SILC includes a variable for the type of household which classifies single-parent households as those where only one adult and at least one child live, Chzhen and Bradshaw (2012) indicate that this variable does not allow the identification of single parent who live with their children in households of extended families. Therefore, similarly to these authors, we have defined households of single-parent mothers all of those where there are mothers who do not have a partner, regardless whether there are other adults living in the home. On the other hand, two-parent households are defined as those where parents live as a couple.

Secondly, we include variables on the educational attainment of parents. In the case of two-parent homes, these variables indicate the highest level of education achieved by any of the parents (if different). Following Garriga and Berta (2018), we distinguish between three different categories of education: high educational level (tertiary education), medium educational level (upper secondary education or post-secondary education) and low educational level (without education, primary education or lower secondary education).

Apart from the main explanatory variables, and in a similar way to previous works, we also include a set of control variables (Bárcena-Martín et al., 2017a, b; Chzhen & Bradshaw, 2012). Firstly, we consider some characteristics of parents such as age (classified in four categories according to the age of the household’s oldest parent: older than 50, between 41 and 50, between 31 and 40, and 30 or younger) and country of birth (immigrant and non-immigrant parents). We also use variables that describe household characteristics. We measure the proportion of active members who are unemployed through a continuous variable of unemployment intensity in the household. We take into account the number of children (grouped in two categories: fewer than three children and three children or more), the age of the youngest child (younger than 12 and between 12 and 15), the household annual equivalent disposable income (grouped by quintile)^5^ and the density of the population in the area where the household is located (area with a high population density and area with low or medium population density).

### Analytic Strategy

Our main analysis is based on the estimation of logistic regression models, given that the dependent variables for child deprivation are binary variables. We use the same strategy as previous works which have examined the differential effects of family structure according to the socioeconomic profile of parents on different measures for child well-being (Albertini & Dronkers, 2009; Garriga & Berta, 2018; Mandemakers & Kalmijn, 2014). In particular, for each of the dependent variables, and for the Spanish and Italian cases separately, we estimate two types of models. In the first, we include variables for family structure and educational attainment of parents, as well as the rest of independent control variables, with the objective of analysing their relationship with different types of material child deprivation.^6^ In the second, we add interactions between the family structure variable and the educational level variables. Through this last model, we seek to analyse if the link between single parenthood and child deprivation varies according to the educational level of mothers.

## Results

Before presenting the results of the logistic regression models, we provide a brief description of the two types of family analysed. Firstly, Table 1 shows the percentages of two-parent families and single-parent families headed by mothers in Italy and Spain. The data is quite similar for the two countries, with the percentage of two-parent families (88% in Italy and 87% in Spain) much higher than single-parent families (12% in Italy and 13% in Spain).

**Table 1:** Percentages of family types, 2014

	Italy	Spain
Two-parent family	88.49	87.46
Single-mother family	11.51	12.54

*Source*: EU-SILC 2014 cross-sectional data

The data are for those households with at least one child between 1 and 15 years old

Table 2 shows the educational structure of the two types of families. On the whole, the data indicates that single-parent families headed by mothers have less educational attainment than two-parent families, particularly in Spain. More specifically, in Italy the percentage of two-parent families where the parents have low education is 22%, while the percentage of single-parent mothers with the same level is 27%. In the case of Spain, 25% of two-parent families have parents with low educational attainment, as opposed to 43% of single-parent mothers. In Italy percentages of two-parent and single-mother families where parents have a medium educational level are quite similar (51% and 50% respectively). There is also no great difference between the percentages for these types of families in Spain (24% for two-parent and 22% for single-mother families). Lastly, in both Italy and Spain, the percentage of two-parent families with high educational attainment parents is greater than that of single-parent mothers with the same level of education. However, in Italy the difference is much smaller (27% of two-parent families as opposed to 23% of single-parent mothers) than in Spain (51% of two-parent families and 36% of single-parent mothers).^7^

**Table 2:** Parents’ educational level by family types (percentages), 2014

	Italy		Spain	
	Two-parent family	Single-mother family	Two-parent family	Single-mother family
Low educational level	22.32	26.78	25.48	42.55
Medium educational level	50.72	49.98	23.62	21.65
High educational level	26.96	23.24	50.90	35.80

*Source*: EU-SILC 2014 cross-sectional data

The data are for those households with at least one child between 1 and 15 years old

Table 3 shows the rest of the characteristics of the parents and households. As can be seen, in Italy and Spain the percentage of single-parent mothers in the youngest age groups is higher than the percentage of parents from two-parent families. Likewise, the percentage of immigrant single-parent mothers is also higher. For both countries, the unemployment intensity in single-mother families is higher than in two-parent families. On the other hand, in single-mother families there are fewer children, and the youngest child is more likely to be older. The data also shows that these families have less income and a greater tendency to live in densely populated areas than two-parent families.

**Table 3:** Parents and household characteristics according to family type, 2014

	Italy		Spain	
	Two-parent family	Single-mother family	Two-parent family	Single-mother family
Parent's age (%)				
Older than 50 years old	14.93	5.60	13.04	6.16
41–50	52.92	41.96	50.10	40.14
31–40	29.97	44.33	34.97	41.33
30 years old or younger	2.18	8.11	1.89	12.37
Immigrant parents (%)	12.24	14.37	14.23	22.54
Household unemployment intensity (mean)	0.12	0.17	0.24	0.30
Household with three or more children (%)	9.01	6.13	9.60	4.79
Age of the youngest child 12–15 (%)	19.69	26.32	19.40	27.56
Household income quintile (%)				
Q5	12.92	5.31	18.34	8.82
Q4	18.70	8.76	17.91	13.76
Q3	19.64	13.90	19.18	16.39
Q2	22.41	26.70	19.77	22.70
Q1	26.33	45.33	24.80	38.33
Densely populated area (%)	42.30	52.56	49.02	53.89

*Source*: EU-SILC 2014 cross-sectional data

The data are for those households with at least one child between 1 and 15 years old

Table 4 shows the results of the first type of model proposed. Firstly, the results indicate that family structure is not linked to any of the five types of child deprivation in Italy, whilst in Spain it does have an impact on material deprivation of education.^8^ In particular, children of single-parent mothers in Spain are more at risk to being exposed to deprivation in this dimension of child well-being in comparison with children from two-parent households. On the other hand, the educational level of parents is seen to be a determinant factor for deprivation in both countries, as children of parents with a low level of education are more likely to suffer deprivation in any of the five dimensions studied. Moreover, children of parents with medium education attainment also have a higher probability of suffering educational deprivation in Italy and the five types of deprivation in Spain.

**Table 4:** Logistic regressions of child deprivation

	Italy					Spain				
	Nutrition	Clothing	Education	Leisure	Social life	Nutrition	Clothing	Education	Leisure	Social life
Single-mother family *(ref.: Two-parent family)*	0.016	0.009	-0.098	-0.180	-0.203	-0.017	-0.187	0.785***	0.104	0.044
Parent's educational level *(ref.: High level)*										
Medium level	0.204	-0.297	0.231*	0.077	0.157	0.469*	0.464*	0.692***	0.325**	0.468**
Low level	0.545***	0.639***	0.818***	0.522***	0.628***	0.795***	0.587**	0.723***	0.667***	0.632***
Parent's age *(ref.: Older than 50 years old)*										
41–50	-0.208	-0.428**	-0.064	0.150	-0.011	-0.281	0.165	-0.315	-0.120	-0.231
31–40	-0.406*	-0.444*	0.084	0.530***	0.343**	-0.049	-0.031	-0.230	0.363*	0.446**
30 years old or younger	-0.553	-0.503	0.215	1.045***	0.682**	-0.837	0.007	-0.332	0.992***	0.586*
Immigrant parents *(ref.: Non-immigrant parents)*	0.531***	0.858***	0.711***	0.573***	0.474***	0.362	0.477**	0.378*	0.641***	0.788***
Household unemployment intensity	0.286	0.633***	0.704***	0.731***	0.748***	1.182***	1.362***	1.130***	1.017***	1.190***
Household with three or more children *(ref.: Less than three children)*	0.214	0.470**	0.285*	-0.181	-0.189	0.559**	0.800***	1.189***	0.426**	0.434**
Age of the youngest child 12–15 *(ref.: 0–11)*	0.140	-0.313	0.128	0.039	-0.059	0.291	-0.396	0.206	0.204	0.261
Household income quintile *(ref.: Q5)*										
Q4	0.692***	1.122***	0.644***	0.286*	0.501***	0.573	2.208**	-0.046	0.463	0.343
Q3	0.429*	0.806**	0.974***	0.488***	0.736***	0.533	2.762***	0.251	0.385	0.656*
Q2	0.903***	0.964***	1.243***	0.750***	0.773***	0.557	2.811***	0.903*	1.008***	1.025***
Q1	1.063***	1.539***	1.721***	1.333***	1.274***	1.152**	3.736***	1.113**	1.234***	1.653***
Densely populated area *(ref.: Non-densely populated area)*	-0.199	-0.090	-0.107	-0.140	-0.134	-0.044	0.337*	-0.089	0.220*	0.121
Constant	-2.747***	-3.014***	-2.383***	-1.583***	-2.198***	-4.350***	-6.831***	-3.729***	-2.920***	-3.535***
Observations	4160	4160	4160	4160	4160	3066	3066	3066	3066	3066
Pseudo R^2^	0.046	0.102	0.119	0.094	0.080	0.120	0.198	0.171	0.151	0.201

*Source:* EU-SILC 2014 cross-sectional data

* p < 0.10; ** p < 0.05; *** p < 0.01

Regarding the control variables, the results show that children whose parents are aged under 40 are more likely to suffer deprivation in leisure and social life with respect to older parents in both countries. Nevertheless, in the case of Italy, children of parents aged between 31 and 40 are less likely to suffer deprivation in nutrition, and those of parents between 31 and 50 have a lower risk of deprivation in clothing. In so far as the parents’ country of birth is concerned, in Italy children of immigrant parents have a higher risk of suffering any of the five types of child deprivation. In the case of Spain, the deprivation risk in all dimensions is higher for these children, with the exception of nutrition, for which no significant differences are observed with respect to children of non-immigrant parents. The higher the intensity of unemployment is in the household, the greater the probability of children being exposed to deprivation in all five dimensions, although in Italy this result is not observed for deprivation in nutrition. In Italian families with three or more children there is an increased risk of child deprivation in clothing and education. For their part, Spanish households with more than two children have a higher risk of child deprivation in all dimensions. The age of the youngest child does not seem to affect child deprivation in either country. It is also confirmed that, in general, households with a lower income have a higher risk of child deprivation in all studied dimensions in both countries. Finally, population density is not shown to have an impact on child deprivation in Italy, while Spanish children living in more densely populated areas are more likely to experience deprivation in clothing and leisure.

**Fig. 2:**
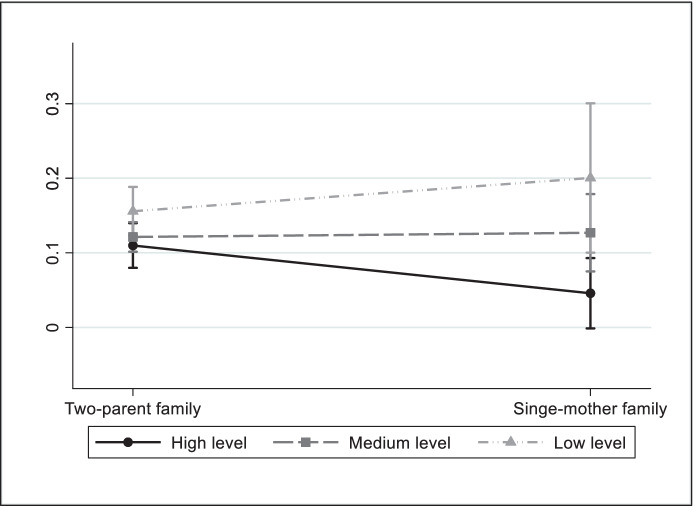
Predicted probabilities of child deprivation in nutrition by family structure and parents' educational level, Italy. Based on Table 5

**Fig. 3:**
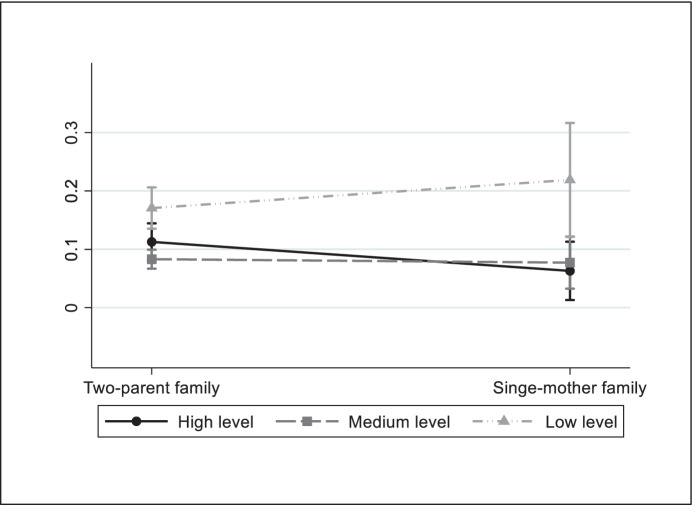
Predicted probabilities of child deprivation in clothing by family structure and parents' educational level, Italy. Based on Table 5

Table 5 shows models which include interactions between family structure and parents’ educational level. In Italy, of the five types of deprivation analysed, the results show that the coefficient of the interaction between family structure and the low educational level variable is significant (with a positive sign) for deprivation in nutrition and clothing. To facilitate interpretation, these results are represented graphically through predicted probabilities in Figs. 2 and 3. As Fig. 2 shows, the predicted probability of suffering deprivation in nutrition is greater amongst children of single-parent mothers with a low educational level than in two-parent families with the same level of education. Family structure does not seem to have any effect on deprivation of children whose parents have a medium educational level. In contrast, the likelihood of suffering nutritional deprivation is minor for children of single-parent mothers with a high educational level. Figure 3 shows that the probability of child deprivation in clothing is greater amongst children of single-parent mothers with a low educational level than those of two-parent families. With regards to the other three types of child deprivation, the results in Table 5 do not show a differential effect for the type of family according to the educational attainment of parents.

**Table 5:** Logistic regressions of child deprivation. Interactions between family structure and parents' educational level

	Italy					Spain				
	Nutrition	Clothing	Education	Leisure	Social life	Nutrition	Clothing	Education	Leisure	Social life
Single-mother family *(ref.: Two-parent family)*	-0.961*	-0.667	0.133	-0.363	-0.540	0.318	-0.153	0.220	0.088	-0.769*
Parent's educational level *(ref.: High level)*										
Medium level	0.115	-0.357*	0.288**	0.042	0.133	0.461	0.387	0.574**	0.343**	0.365*
Low level	0.414*	0.515**	0.812***	0.516***	0.538***	0.896***	0.652**	0.607***	0.645***	0.497***
Single-mother family x Parent's educational level										
Single-mother family x Medium level	1.014	0.584	-0.482	0.318	0.218	0.052	0.573	0.740	-0.142	0.968*
Single-mother family x Low level	1.277*	1.001*	0.027	0.074	0.709	-0.639	-0.380	0.683	0.111	1.080**
Constant	-2.668***	-2.951***	-2.413***	-1.564***	-2.162***	-4.395***	-6.857***	-3.677***	-2.912***	-3.481***
Observations	4160	4160	4160	4160	4160	3066	3066	3066	3066	3066
Pseudo R^2^	0.048	0.104	0.120	0.095	0.081	0.122	0.200	0.172	0.151	0.203

*Source*: EU-SILC 2014 cross-sectional data

The other independent variables shown in Table 3 are also included in the estimations. ^*^ *p* < 0.10; ^**^ *p* < 0.05; ^***^ *p* < 0.01

**Fig. 4:**
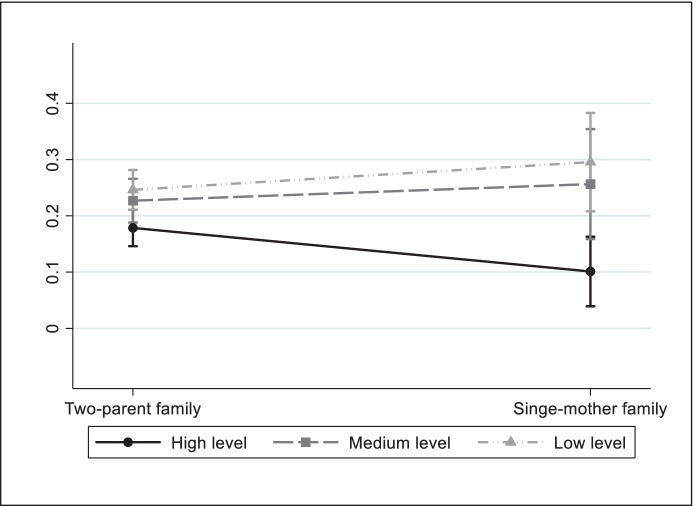
Predicted probabilities of child deprivation in social life by family structure and parents' educational level, Spain. Based on Table 5

In the case of Spain, coefficients of interactions between family structure and the low educational level and medium educational level variables are significant (with a positive sign) for child deprivation in the social life dimension. As we show in Fig. 4, the predicted probability of social life deprivation is greater for children of single-parent mothers with a medium and low educational level than those of two-parent families with the same level of qualifications. On the other hand, the probability of deprivation in this dimension is smaller for children of single-parent mothers with a high educational level.

## Conclusion and Discussion

This study aims to deepen our understanding of the effect of single parenthood on child well-being in southern Europe by introducing different indicators of child deprivation. We have focused on Italy and Spain, examining the link between single parenthood and child deprivation, and if the latter differs according to the educational attainment of mothers. In order to measure child deprivation, we follow the EU-MODA approach which allows us to distinguish between deprivations in nutrition, clothing, education, leisure and social life. This distinction gives us the possibility to identify the dimensions of deprivation that are most affected by family transformations in Italy and Spain.

First of all, when we analyse the effect of single parenthood without differentiating the educational profile of mothers, the results reflect that in Italy children of single-parent mothers do not have a higher risk of material deprivation with respect to children of two-parent families in any of the study’s dimensions. This is due to the fact that the difference in economic resources between the two types of families is not very big in south European countries (Hampden‐Thompson, 2009). However, in the case of Spain, children of single-parent mothers do have a greater risk of deprivation in the education dimension. This result suggests that these children have, for example, fewer suitable books for their age, or participate less frequently in school trips than the children of two-parent families. The higher probability of deprivation in education in single-parent families may be linked to the fact that Spain is one of the Mediterranean countries where acceptance of new family models and lifestyles, such as two-parent families with two breadwinners, has increased most (Moreno & Marí-Klose, 2013; Naldini & Jurado, 2013). Thus, the differences in resources between single-parent and two-parent families may be growing in Spain, with consequences for inequality in child well-being (Nieuwenhuis & Maldonado, 2018; Waldfogel et al., 2010).

Regarding the relation between family structure and child deprivation according to the educational level of parents, we found evidence of the detrimental effect of single parenthood in families with fewer educational resources. In particular, in the case of Italy we observed that children of single-parent mothers with a low educational level have an increased risk of deprivation in nutrition and clothing than those of two-parent families. Therefore, in this country the effect of single parenthood is concentrated in households headed by mothers with few qualifications. This may possibly be explained by the fragile economic situation of these families as a consequence of the difficulties that mothers with smaller human capital experience in integrating into the job market (Härkönen et al., 2016; Van Damme et al., 2009). In fact, children of single-parent mothers with higher qualifications have a reduced risk of nutrition deprivation than children of two-parent families. In the case of Spain, our findings indicate that children of single-parent mothers with medium or low educational level are at a higher risk of deprivation in the social life dimension, in comparison with those of two-parent families. On the contrary, children of single-parent mothers with a high educational attainment have a lower probability of deprivation in this dimension. Given that social life deprivation implies not celebrating special occasions, such as birthdays, or not inviting the children’s friends to play or eat at home, the results obtained may reflect that in Spain single-parent mothers with less educational attainment do not dedicate enough time to their children, which in turn has a negative impact on children’s socialization skills (Ridge, 2007; Ridge & Millar, 2011). Even though the development of family policies in Spain during the last few decades has facilitated the insertion of women into the labour market, this in itself may not have been enough to solve problems of work and family life reconciliation experienced by mothers with fewer qualifications (Crespi & Moreno Mínguez, 2017).

Overall, our findings are in line with those of previous studies that show that living in single-parent households is particularly detrimental for children whose mothers have less education (Albertini & Dronkers, 2009; Augustine, 2014; Garriga & Berta, 2018; Grätz, 2015; Mandemakers & Kalmijn, 2014). Nevertheless, it should also be pointed out that we did not find that single parenthood in families in worse socioeconomic positions affects all types of child deprivation. In fact, deprivation indicators which are seen to be affected are not the same in Italy and Spain, which may be related to the different work and family reconciliation policies in place in each of these countries (Crespi & Moreno Mínguez, 2017; Naldini & Jurado, 2013).

In any case, this study shows that the emergence of the negative educational gradient of single motherhood is a fundamental factor for the growth of child well-being inequality in the south of Europe. The consequences of this situation are reflected in the increase of child deprivation, not only in economic terms but also in access to participatory and social resources that contribute to developing cognitive and personal skills. The case of Spain is especially relevant due to the fact that, as shown by our descriptive results and other previous research (Garriga & Cortina, 2017; Garriga et al., 2015), the prevalence of single-parent mothers with a low educational profile is greater there than in Italy.

Lastly, the results obtained also confirm that parents’ age and nationality, unemployment intensity, number of children and household income are determinants of child poverty. These findings are quite similar to those of other previous research carried out in the context of Europe (Bárcena-Martín et al., 2017a, b; Chzhen & Bradshaw, 2012).

Although our analysis focused on a moment in time when the most disadvantaged families were still much affected by the economic and employment aftermath of the 2008 crisis, future studies will have to explore the impact of the Covid-19 pandemic on inequality according to type of family and educational attainment of parents. In contexts such as the Covid-19 crisis, single-parent mothers with a low educational level face a greater risk of loss of employment and income. Consequently, the children of these mothers are in a very vulnerable position in southern Europe, where the labour and economic impact of successive crises has been very intense (Duell et al., 2016; Fana et al., 2020). Family policies aimed at the specific needs of single-parent families have been practically non-existent in Italy and Spain (Almeda et al., 2016; Van Lancker et al., 2015), increasing the precariousness of these families. This research has shown that the concentration of single-parent families among women with less education has contributed to increasing child inequality. Public policies should put this empirical evidence of inequality in child deprivation in context with the implementation of specific policies aimed at improving the situation of single-parent families with fewer resources and contributing to child well-being.

In short, the sociodemographic changes produced in families due to the individualizing process of SDT increases the gap between families in parental resources and styles, which has repercussions on inequality and the reproduction of material and sociocultural deprivation of children (Garriga et al., 2015; McLanahan, 2004). If the increase of single parenthood presented new challenges for Mediterranean welfare states, the assertion that the negative incidence of educational level may further accentuate them suggests that family policies in Italy and Spain may not be sufficiently developed to confront the needs arising from these family and social changes.

## Appendix 1

Tables 6 and 7

**Table 6:** Parents' educational level by family types (percentages), 2018

	Italy		Spain	
	Two-parent family	Single-mother family	Two-parent family	Single-mother family
Low educational level				
	24.21	26.91	21.13	38.87
Medium educational level				
	46.18	48.34	22.90	25.48
High educational level				
	29.62	24.75	55.98	35.65

*Source*: EU-SILC 2018 cross-sectional data.

The data are for those households with at least one child between 1 and 15 years old.

**Table 7:** Logistic regressions of child deprivation (without control variables)

	Italy					Spain				
	Nutrition	Clothing	Education	Leisure	Social life	Nutrition	Clothing	Education	Leisure	Social life
Single-mother family *(ref.: Two-parent family)*	0.088	0.187	0.240*	0.223*	0.150	0.245	0.141	0.912***	0.556***	0.521***
Constant	-1.926***	-2.075***	-0.816***	-0.442***	-1.015***	-2.695***	-2.449***	-2.128***	-1.092***	-1.352***
Observations	4160	4160	4160	4160	4160	3066	3066	3066	3066	3066
Pseudo R^2^	0.000	0.001	0.001	0.001	0.000	0.001	0.000	0.018	0.006	0.006

*Source:* EU-SILC 2014 cross-sectional data.

* p < 0.10; ** p < 0.05; *** p < 0.01.

## Footnotes

1 We exclude widow mothers, as a great part of the literature on the educational gradient of single-parent mothers does not usually encompass this group (Härkönen, 2017). Nevertheless, for the sake of robustness, we have included these mothers in alternative analyses, with the main results remaining practically unaltered.

2 In regression analysis, the household is used as an observation unit given that, as Bárcena-Martín et al. (2018) point out, children of the same household are not statistically independent observations.

3 The information about mandatory school age in Italy and Spain was obtained from the European Commission (2014b).

4 The information about attendance to nurseries is not part of the child deprivation module for 2014, as EU-SILC provides it on an annual basis. In additional analyses (available on request) we did not include this indicator in education deprivation. The results obtained are similar to those presented in this study.

5 The EU-SILC variable of income informs about income obtained in the year previous to that of the interview; hence, we are controlling for the income obtained in 2013. It should be noted that this income variable only offers partial information on household economic resources, which depend on past and present income, accumulated savings, investments in durable goods, or financial support from friends and family (Guio et al., 2020; Verbunt & Guio, 2019).

6 Models where independent variables are included step-by-step are available on request.

7 Table 6 in the Appendix provides more recent data on the parents' educational level (year 2018). In general, the differences in educational level between single-mother and two-parent families are quite similar to those of 2014.

8 The relationship between family structure and child deprivation without controlling for the rest of parent and household characteristics is shown in Table 7 in the Appendix.
